# Using a novel assessment of procedural proficiency provides medical educators insight into blood pressure measurement

**DOI:** 10.5116/ijme.580b.2e4f

**Published:** 2016-11-19

**Authors:** Michael Holmstrup, Brock Jensen, Rebecca Burkart, Malorie Levis

**Affiliations:** 1 Department of Exercise and Rehabilitative Sciences, Slippery Rock University, Slippery Rock, PA, USA

**Keywords:** Think aloud, instructional methods, interdisciplinary medical education, qualitative research methods

## Abstract

**Objective:**

This investigation was performed to determine how students in a health sciences program utilize and explain techniques within blood pressure measurement using a novel assessment, and changes associated with greater curricular exposure.

**Methods:**

An exploratory, qualitative and quantitative study was conducted using a ‘Think Aloud’ design with protocol analysis. Following familiarization, participants performed the task of measuring blood pressure on a reference subject while stating their thought processes. A trained practitioner recorded each participant’s procedural proficiency using a standardized rubric. There were 112 participants in the study with varying levels of curricular exposure to blood pressure measurement.

**Results:**

Four trends are noted. Specifically, a trend was observed wherein a marked increase in procedural proficiency with a plateau occurred (e.g. released cuff pressure 2-4 mmHg, 10%, 60%, 83%, 82%). Secondly, a trend was observed with improvement across groups (e.g. cuff placed snugly/smoothly on upper arm, 20%, 60%, 81%, and 91%). Other trends included a marked improvement with subsequent decrease, and an improvement without achieving proficiency (e.g. palpation of the brachial pulse, 5%, 90%, 81%, 68%, appropriate size cuff, 17%, 40%, 33%, 41%, respectively). Qualitatively, transcript interpretation resulted in a need for clarification in the way blood pressure procedure is instructed in the curriculum.

**Conclusions:**

The current investigation provides a snapshot of proficiency in blood pressure assessment across a curriculum and highlights considerations for best instructional practices, including the use of Think Aloud. Consequently, medical educators should use qualitative and quantitative assessments concurrently to determine achievement of blood pressure skill proficiency.

## Introduction

Blood pressure (BP) is one of the most commonly measured vital signs used to assess an individual’s cardiovascular health.^1^ Specifically, auscultation using a sphygmomanometer and stethoscope remains the gold standard method of BP measurement. A BP measurement less than 120/80 mmHg is classified as normal, while measurements ≥120/80 mmHg can result in other classifications (e.g. prehypertension and hypertension) known to be closely linked to increased risk for morbidity and premature mortality.^2^

Despite the prevalence and importance of measurement in the health field, BP is often measured inaccurately. It has been suggested that failure to accurately measure BP in a clinical setting is related to 3 main reasons: 1) methodological inaccuracy (e.g. inappropriate cuff size, arm position, rapid deflation of cuff, etc.), 2) the inherent variability of BP, and 3) the so called ‘white-coat’ effect.^3^ Although inherent limitations to arterial BP measurement exist, improving the practitioner’s skill in BP measurement would be a practical approach to minimizing inaccuracies that may lead to the misclassification of normotensive, prehypertensive, and/or hypertensive individuals.^3^^,^^4^

Furthermore, proficiency in BP measurement is an important safety measure for clinical professionals who use exercise as an intervention to improve or rehabilitate health-related physical fitness.^3^^,^^4^ In light of this need for proficiency, the curricula implemented to train health care professionals need to adequately prepare students in the accurate use of skills associated with BP measurement.  

The physical skills associated with BP measurement have been highlighted in the American Heart Association Practice Guidelines.^5^ The clinical professional must have proficiency in both the setup (e.g. arm supported at heart level, upper arm bared, cuff bladder encompassing ≥80% of arm circumference, etc.) and measurement skills (mercury column deflated 2-4 mmHg/sec, first and last audible sounds recorded as systolic and diastolic measures, etc.) associated with the quantification of BP.^5^ While greater attention has been placed on physical skills required for valid BP measures, to our knowledge, no investigations have attempted to understand the underlying thought processes that accompany BP measurement. Recently, the ‘Think Aloud’ (TA) method has been used to characterize an individual’s thought processes during the performance of a concurrent task.^6^^,^^7^ These observations have led to recommendations for improvement in task performance and pedagogy.^6^^,^^7^ Thus, it is plausible that combining both physical and cognitive observations may reveal a more comprehensive view of common practitioner strengths and weaknesses in BP measurement and may lead to valuable recommendations for instructional practice.

Therefore, the purpose of this study was to observe both procedural proficiency and cognitive processes of undergraduate students at various levels of an Exercise Science curriculum as they perform a BP measurement.

## Methods

### Participants and sample size     

In the United States, undergraduate Exercise Science programs generally consist of four years. In the present study, undergraduate Exercise Science students at Slippery Rock University were recruited from four distinct levels of the curriculum. As noted, curricular exposure refers to hours devoted to a given topic in an Exercise Science course at Slippery Rock University. Group (BP-0) consisted of students in the pre-200 level courses, with zero hours of curricular exposure to BP technique. Group (BP-4) consisted of students who completed a 200-level Exercise Physiology course, and had 4 hours of curricular exposure (supervised BP instruction and practice), and one practical examination on resting BP measurement. Group (BP-10) students had completed an additional 400-level Fitness Assessment course, which included 10 additional hours of curricular exposure (supervised BP practice; 14 hours total) and an additional practical examination on BP measurement during exercise. Group (BP-14) students were in a 410-level capstone course (14 total hours of curricular exposure, two practical examinations) which provided no additional BP technique instruction but required students to implement BP measurement during assessment and exercise monitoring with an actual client.

Prior to any data collection, procedures were approved by the Slippery Rock University Institutional Review Board to ensure maintenance of ethical standards. All participants completed an informed consent document prior to completing the study, and ethical regulations were obeyed.

### Data collection methods

A procedural rubric (Appendix 1) was created in order to collect data on the use of accepted procedural steps^4^ in the appropriate measurement of blood pressure. Further, the ‘Think Aloud’ method, where the study participant verbalizes their thoughts while performing a concurrent task, was utilized. The variables observed in this study were quantifications of PP (procedural proficiency) and TA (‘Think Aloud’) during the act of blood pressure measurement at four distinct levels of our curriculum.

### Procedure

At the start of each data collection session, the participant was oriented to the TA method by a trained researcher who read from a standardized script. Briefly, as the process is described elsewhere,^7^each participant completed a mathematical problem and solved an anagram while verbally expressing all of the thoughts that were going through their conscious mind during the completion of the task. The researcher was not interested in a correct answer, rather, the amount of information and level of detail expressed by the participant in relating their thoughts. Following this exposure, the researchers oriented the participant to the observational/TA task with the following consistent introductory statement:

“I would like you to perform a blood pressure measurement on the subject. I will present you with the subject, and you will perform this task to the best of your ability. Please talk aloud as you perform this blood pressure assessment. Remember, if you are silent for any length of time I will remind you to keep talking aloud.”

Following the orientation, each participant completed a BP measurement while implementing the TA method. Each session was taped using a digital recording device. The only time that the researchers provided any feedback during the BP measurement was to remind the participant to continue ‘talking aloud’ if they fell silent for any length of time.

Each BP measurement was performed using the standard auscillatory method (sphygmomanometer and teaching stethoscope) on a consistent, normal-weight, female test subject. During the measurement, a trained researcher took notes on a standardized procedural rubric (Table 1) regarding the participant’s BP technique, while another trained researcher listened in on the teaching stethoscope with the participant to determine a BP value. The researcher and subject then recorded their BP measurements independently.

### Data analysis

The percentage of procedural proficiency (PP) for each associated technique was calculated using the sum of observed participants implementing the technique divided by the total number of participants in the curriculum cohort. For example, 9 out of 22 participants in BP-14 correctly identified an appropriately-sized BP cuff to use for the test subject (PP- 41%).

**Table 1:** Proportions of observed procedural proficiency and mentioned thoughts transcribed using ‘Think Aloud’ during sphygmomanometer set-up, n=112, Slippery Rock University 2015

Procedural proficiency	BP-0		BP-4		BP-10		BP-14	
	PP^*^ (%)	TA^†^ (%)	PP (%)	TA (%)	PP (%)	TA (%)	PP (%)	TA (%)
Palpated for brachial pulse	5	19	90	77	81	94	68	100
Appropriate-sized cuff	17	8	40	69	33	91	41	100
Cuff smoothly/snugly on arm	20	14	60	77	81	79	91	86
Cuff on skin, not clothing	78	38	50	62	83	73	86	86
Cuff 1" above antecubital	7	3	60	8	92	3	95	14
Centre of bladder over artery	7	14	50	62	100	73	95	86

*PP: Procedural Proficiency; †TA: ‘Think Aloud’

All of the digital TA files recorded during BP measurement were converted into written transcripts. Two investigators used a randomly-selected small sample of these transcripts (n=6) to inductively develop a coding rubric to classify the strategies used by participants during the BP exercise in line with previously published work.^6^^,^^7^ Following the development of this rubric, the investigators independently coded an additional small set (randomly selected) of the TA transcripts (n=6) using this new rubric. Upon achieving an inter-coder agreement coefficient >85%,^6^^,^^7^ small changes were made for clarification, and then all of the participant transcripts were coded by an independent investigator. When a participant correctly mentioned a strategy during the measurement, this was noted. In line with our previous example, 22 out of 22 participants in BP-14 correctly mentioned the need to choose an appropriately-sized BP cuff for the test subject (TA- 100%). Comparisons between the strategies and procedures implemented (PP) and mentioned (TA) by the participants at the four distinct levels of the curriculum are reported qualitatively.

**Table 2:** Proportions of observed procedural proficiency and mentioned thoughts transcribed using ‘Think Aloud’ during stethoscope set-up, n=112, Slippery Rock University 2015

Procedural proficiency	BP-0		BP-4		BP-10		BP-14	
	PP^*^ (%)	TA^†^ (%)	PP (%)	TA (%)	PP (%)	TA (%)	PP (%)	TA (%)
Ear pieces forward	24	0	50	23	100	58	95	29
Stethoscope on	0	11	50	38	67	67	59	14

*PP: Procedural Proficiency; †TA: ‘Think Aloud’

**Table 3:** Proportions of observed procedural proficiency and mentioned thoughts transcribed using ‘Think Aloud’ during measurement, n=112, Slippery Rock University 2015

Procedural proficiency	BP-0		BP-4		BP-10		BP-14	
	PP^*^ (%)	TA^†^ (%)	PP (%)	TA (%)	PP (%)	TA (%)	PP (%)	TA (%)
Diaphragm side	76	0	100	8	92	30	100	0
Diaphragm on artery	29	8	70	38	100	55	95	43
Diaphragm solid contact	49	3	90	15	100	39	95	43
Arm straight	59	11	50	23	100	58	95	29
Arm at heart level	32	5	70	15	78	52	82	71
Shut valve, inflate ~30mmHg	17	27	80	62	92	52	95	0
Release 2-4mmHg/second	10	5	60	8	83	42	82	29
SBP^‡^ at phase 1 Korotkoff	0	19	10	77	58	91	41	57
DBP^¶^ at/before phase 5	0	16	10	77	58	91	45	57
After DBP, deflate rapidly	7	0	40	0	83	21	95	0
Report BP w/even numbers	5	14	40	77	86	91	91	57

*PP: Procedural Proficiency; †TA: ‘Think Aloud’; ‡SBP: systolic blood pressure; ¶DBP: diastolic blood pressure

## Results

Subjects were assigned to one of four primary groups: BP-0 (n=41), BP-4 (n=13), BP-10 (n=36), and BP-14 (n=22) based on their placement in the Exercise Science curriculum. Data collected from each group were assessed independent of the other groups. This process involved separating the data by each variable listed on the standardized rubric (Appendix 1; e.g. palpated for brachial pulse). Looking at each variable independently, the percentage of students in each group who successfully completed the skill was determined. The results from each group were then compared among the four groups (BP-0, BP-4, BP-10, and BP-14).  If 70% of students successfully completed a skill, the cohort was considered proficient. This percentage of proficiency was selected because it is a commonly used percentage of proficiency in nursing education by organizations including the Ohio Nurses Association and the American Nurses Credentialing Center’s Commission on Accreditation.^8^

### General observations

Generally, PP and TA were below the set 70% proficiency criterion in BP-0 (Tables 1-3). Both PP and TA revealed there was a progressive improvement with a greater curricular exposure. With regards to selecting the appropriate cuff, PP was revealed as ≤ 41% for all groups. TA for selecting the appropriate cuff, however, resulted in 91% of BP-10 and 100% of BP-14. TA for cuff placement 1 inch from the antecubital fossa resulted in 3% and 14%, in BP-10 and BP-14, respectively. PP for cuff placement resulted in 92% and 95%, in BP-10 and BP-14, respectively. Stethoscope set-up resulted in low PP and TA in BP-0 and BP-4 ≤ 50%. Task variables indicative of measurement, such as properly selecting the diaphragm side, placing the diaphragm with solid contact over the artery, holding the arm at heart level, and proper inflation of the cuff all resulted in acceptable PP (≥70% in groups BP-4, BP-10 and BP-14).

### Procedural proficiency

Four distinct trends were observed in the data.  Specifically, a trend was observed from BP-0 through BP-14 wherein a marked increase in PP with a plateau occurred (e.g. released cuff pressure 2-4 mmHg, 10% to 60% to 83 to 82%, Table 3).  Secondly, a trend was observed with continual improvement from BP-0 through BP-14 (e.g. cuff placed snugly/smoothly on upper arm, 20% to 60% to 81% to 91%, Table 1). Other trends included a marked improvement with subsequent decrease, and an improvement with low proportion of proficiency (e.g. palpation of the brachial pulse, 5% to 90% to 81% to 68%; appropriate size cuff used, 17% to 40% to 33% to 41%; respectively, Table 1). The most common trend observed was the improvement with plateau.

### ‘Think Aloud’

Similar to PP, common trends from groups BP-0 through BP-14 included a marked increase with a plateau (e.g. cuff placed snugly/smoothly on upper arm, 14% to 77% to 79% to 86%, Table 2), continual improvement (e.g. palpated for brachial pulse, 19% to 77% to 94% to 100%, Table 1), and a marked improvement with subsequent decrease (e.g. using the diaphragm side, 11% to 38% to 67% to 14%, Table 1).

Examples of several transcript excerpts that highlight the importance of ‘appropriate cuff size selection’ in the procedural findings are presented. Differences can be noted between individuals from BP-0 to BP-14, though the expected misunderstanding (in students without dedicated BP instruction) was not completely resolved with the current structure of the BP measurement instruction in place prior to the ‘Think Aloud’ analysis.

###         BP-0

“Does it matter which cuff I pick? Well, you’re not a child, so I’m not going to use the littler one, and you’re not a very large person so I’ll pick the middle one.”

“Let’s see here, that looks kind of small, we will go with the medium.”

###         BP-14

“Hi, I’m ---, first I’m going to measure your arm to know which cuff size to use, so if I could just have your right arm, wait my right, your left (laughter). So once I measure it, okay so it’s 22cm, then I go to different cuffs to look. If I remembered these, I would know which one to use. I pick the small cuff, and then I have to attach it to the sphygmomanometer.”

“I’m going to take your right arm and I’m going to palpate your brachial artery, I’m going to grab a cuff and based on the size of your arm, I’m going to get a regular cuff.”

“I’m going to grab the regular blood pressure cuff, the adult size, and the first thing I’m going to do is find, I’m going to hold your arm under my arm, so it’s at heart level, and I’m going to find the brachial artery, there it is. I’m going to wrap this around here. Does that feel a little loose, tight? I might use the smaller one.”

While the individuals in the BP-14 clearly had an understanding of the need for a specific cuff size for appropriate measurement, and designs on how to achieve the goal of selecting that cuff, their disparate methods led to the selection of the wrong cuff (small cuff was correct) on one of the above referenced, and many of the additional transcripts. This result called attention to a need for clarification in the way that this portion of the BP procedure is instructed in the curriculum, and thus helped to improve outcomes in future instructional sessions. 

## Discussion

Improved proficiency was observed in the majority of procedural skills after the completion of upper-level courses where students were exposed to more teaching, practice, and assessment. This observation is likely the product of our hierarchical program design, where important topics like BP measurement are revisited with increasing depth as students progress through the curriculum. Furthermore, these data suggest that true proficiency in BP measurement may not be achieved without multiple curricular exposures (i.e. supervised instructional sessions and practical assessment). If a health sciences (e.g. Exercise Science, Nursing, Public Health, etc.) curriculum does not provide adequate opportunities that expose students to BP theory and instruct on procedural skills, it is plausible that proficiency may be compromised.

Overall, this study has allowed us to identify the various strengths and pitfalls of all aspects of BP measurement within our curriculum. Our intentional hierarchical design resulted in improvement of 15 of 19 important procedural aspects of BP (i.e. sphygmomanometer set-up, stethoscope set-up, and measurement) by the time students completed the upper-level courses (BP-10 and BP-14). However, that implies a lack of proficiency in 4 of 19 procedural skills. Therefore, those particular skills (appropriate-sized cuff, stethoscope on, SBP at phase 1 Korotkoff, DBP at/before phase 5) need to be addressed more explicitly during instruction within our curriculum.

Interestingly, though proficiency was met, minimal gains were observed between the upper-level courses (improvement with plateau). While there appears to be no further improvement in the percentage of students who were proficient in these skills between these upper-level courses, we found it encouraging that the percentage of proficient students remained constant. This demonstrated that the capstone students who were applying these skills to real clients were maintaining their proficiency without further instruction.

Even though these findings are generalizations from our curriculum, they provide valuable insight into other health sciences curricula regarding BP proficiency. For example, procedural (e.g. appropriate cuff sizing, attention to sphygmomanometer set-up) and cognitive (e.g. mentioning placement of cuff 1” above antecubital space) aspects of our findings may have broad application across the health science disciplines. Further, excerpts from the ‘Think Aloud’ activities allowed insight into the specifics of instructional issues (e.g. why there was an issue with appropriate cuff sizing across the curriculum) and opened doors for addressing these deficiencies (e.g. strict adherence to the measurement of upper-arm circumference and comparison to the 80% rule). While the findings from this investigation allowed our program to confirm specific areas of focus needed in our curriculum, taking the time to conduct a similar analysis in other health science programs may afford additional understanding based on unique aspects (e.g. number of curricular exposure and curricular design, faculty background and training, instructional efficiency) within their educational model. Additionally, future examinations could branch out into other important areas of skill development within the health sciences including medical procedures (e.g. blood draw, catheterization, etc.), field testing (e.g. submaximal and maximal treadmill testing, graded exercise testing, etc.), and bench work (e.g. biological assays, Western blot, etc.). While the undertaking of such analyses would undoubtedly offer useful information regarding program curriculum, this is a time-consuming process with a definite learning curve (particularly in the data analysis aspect).

As such, an observational/TA approach has also been suggested as a means of enhancing skill development within the classroom or lab setting.^7^^,^^9^^,^^10^ In general, skill development requires multiple repetitions and the close supervision of a skilled practitioner. Achieving both of these standards consistently within a classroom setting is not often feasible depending on the number of students and amount of class time dedicated to a particular skill.  A reasonable alternative involves the use of small student groups where individuals assume the roles of client, tester, and observer. In this scenario, the observer (armed with a rubric detailing procedural and cognitive considerations, which could be updated and modified depending on findings) would observe the ‘client/tester interaction’ as the tester practiced the skill using the TA method. Procedural concerns and/or the incomplete explanation of thinking strategies, as previous work has shown that the TA method can identify similar cognitive processes,^11^  could be noted on the rubrics and discussed amongst the small group following the test. The classroom instructor and laboratory assistants could selectively observe and participate in these interactions. Students would be able to discover and reinforce weak points in their procedural proficiency this hands-on setting while developing a better understanding of the cognitive processes underlying proper use of the skill.

A major limitation of the present study is that this observational study was performed within one large academic program at one university, and therefore attempting to generalize specific findings from our curriculum globally may be misguided. Another main limitation of the present study is the specificity of the hierarchical curricular structure presented by our academic program. For example, at other universities in the United States or internationally, various courses or the order in which those courses are presented within a curriculum may vary widely. With this in mind, it is our suggestion that academic units interested in finding specific measurable outcomes for implementation in their curricula perform their own combined analysis with their students. Further, additional procedural steps necessary for blood pressure measurement may have been overlooked by the investigators in this study. Due to the qualitative measure of TA, much of the interpretation of student transcripts was reliant on explicit mention of procedural steps. Therefore, participants could have correctly thought through the act of the blood pressure measure, but failed to communicate this clearly clouding the usefulness of the TA outcome.

## Conclusions

The present findings demonstrate that the combined observational/TA approach provided usable findings for our program in relation to key procedural skills as well as items that undergraduate students should mentally focus on during the completion of BP measurement. Considerations are presented for the use of this promising method for curriculum programming and classroom use. It is emphasized, however, that curricular or programming decisions made based on these methods should be rooted in an extensive piloting and assessment within an academic or clinical program.

### Acknowledgements

The authors wish to extend our gratitude to all of our study participants, as well as our research assistants Jessica McFadden and Tyler Kuhn.

### Conflict of Interest

The authors declare that they have no conflict of interest.

## Supplementary material

Supplementary file 1 Appendix 1. Procedural Rubric for Blood Pressure Measurement
