# Supplementary material for: Using a novel assessment of procedural proficiency provides medical educators insight into blood pressure measurement
Source: Int J Med Educ. 2016 Nov 19;7:375–81. doi: 10.5116/ijme.580b.2e4f (PMC5116368; doi:10.5116/ijme.580b.2e4f)
Supplement: Supplementary file 1 — Appendix 1. Procedural Rubric for Blood Pressure Measurement [file ijme-7-375-S1.pdf]

Appendix 1

Procedural Rubric for Blood Pressure Measurement

	**Y/N**
Sphygmomanometer Set-Up	
palpates for brachial pulse	-----------------------
appropriate-sized cuff	----------------------
cuff is smoothly and snugly around the upper arm	----------------------
cuff was on skin not over clothing	----------------------
cuff is 1 (one) inch above the antecubital fossa	----------------------
centre of the bladder is over the brachial artery	----------------------
Stethoscope Set-Up	
place the ear pieces into ears - ear pieces pointing forward	----------------------
stethoscope head is 'turned' on	----------------------
Measurement	
use the diaphragm side of the stethoscope for measurement	----------------------
stethoscope diaphragm placed over brachial artery	----------------------
stethoscope diaphragm must make solid contact with arm	----------------------
subject's arm is straight	----------------------
arm supported at heart level	----------------------
shut pressure valve and inflated cuff to 30 mmHg above estimated SBP*	----------------------
released air 2-4 mmHg per second or per heart beat	----------------------
SBP is the point at which the first of two or more sounds is heard (phase 1)	----------------------
DBP† is the point before the disappearance of sounds (phase 5)	----------------------
after obtaining the diastolic reading, deflated cuff rapidly and completely	----------------------
reported the blood pressure reading in even numbers (e.g. 124/84 mmHg)	----------------------

*SBP- systolic blood pressure; †DBP- diastolic blood pressure
